# Factors that Influence the Perceived Healthiness of Food—Review

**DOI:** 10.3390/nu12061881

**Published:** 2020-06-24

**Authors:** Brigitta Plasek, Zoltán Lakner, Ágoston Temesi

**Affiliations:** Department of Food Chain Management, Institute of Agrobusiness, Szent István University, Villányi Str. 29-43, 1118 Budapest, Hungary; lakner.zoltan@etk.szie.hu (Z.L.); temesi.agoston@etk.szie.hu (Á.T.)

**Keywords:** perceived healthiness, product attributes, healthy food, consumer perception, food packaging, consumer behavior

## Abstract

The interest of consumers is the consumption of healthy food, whereas the interest of food manufacturers is that consumers recognize the produced “healthier” food items on the shelves, so they can satisfy their demands. This way, identifying the factors that influence the perceived healthiness of food products is a mutual interest. What causes consumers to consider one product more beneficial to health than another? In recent years, numerous studies have been published on the topic of the influence of several health-related factors on consumer perception. This analysis collected and categorized the research results related to this question. This review collects 59 articles with the help of the search engines Science Direct, Wiley Online Library, MDPI and Emerald Insight between 1 January 2014 and 31 March 2019. Our paper yielded six separate categories that influence consumers in their perception of the healthiness of food items: the communicated information—like FoP labels and health claims, the product category, the shape and colour of the product packaging, the ingredients of the product, the organic origin of the product, and the taste and other sensory features of the product.

## 1. Introduction

Which food can be considered beneficial to health? Science and consumers answer this question differently. According to certain sources, there is no precise definition of what can be considered healthy food, or else existing definitions are not yet appropriate [1,2,3]. The understanding of the category of “healthy food” differs even among experts; moreover, some treat the words “healthy” and “nutritious” as synonyms [4,5]. What can be considered healthy for whom depends on gender, age, metabolism, obesity, diseases or sensitivities. A nutritious food product generally considered definitely beneficial to health with several positive effects in case of certain diseases can be harmful for consumers suffering from other diseases [6].

Let us illustrate the effort to define healthy food with two examples. In their article, Zaheer and Bach [7] (p. 1) applied the following definition: “*Per the United States Food & Drug Administration (FDA), Healthy foods are defined as those that are “low in fat, low in saturated fat, contain at least 10% of daily value for vitamins A, C, calcium, iron, protein fiber” and are limited in amount of sodium and cholesterol (USFDA).*” Rodman and his colleagues [5] (p. 83) employed the following definition for their research: *“Foods that provide essential nutrients and energy to sustain growth, health and life while satiating hunger; usually fresh or minimally processed foods, naturally dense in nutrients, that when eaten in moderation and in combination with other foods, sustain growth, repair and maintain vital processes, promote longevity, reduce disease, and strengthen and maintain the body and its functions. Healthy foods do not contain ingredients that contribute to disease or impede recovery when consumed at normal levels. (University of Washington Center for Public Health Nutrition (UWCPHN) 2013* [8])”.

Dieticians argue that there is no such thing as healthy or unhealthy food; instead, there is only appropriate or inappropriate diet (e.g., [1]). However, since consumers consider certain foods healthy, while others unhealthy, it is important for us to know how they make this distinction. Mai and Hoffman [9] (p. 8) use the term perceived healthiness, which, based on Howlett et al. [10], they define as “*Perceived healthiness is a consumer’s expectation of a product’s influence on his or her state of health*”. The importance of “perceived healthiness” is also supported by the research findings on health claims by Steinhauser and colleagues [11] that the higher the level of perceived healthiness of a product is, the more likely it is that the product will be purchased. All this becomes a factor that also increases the willingness to pay and purchase if it takes into account what influences the credibility of the health benefits of a product [12].

The effects of food on health is a widely researched topic, which gets attention from various aspects, thus our knowledge-base related to its consumer perception is also expanding. In their review, Niebylski and colleagues [13] examined the effects of taxation, subsidies and easy access on the consumption of products considered healthy. According to the results of Provencher and Jacob’s review [14] specifically on perceived healthiness, cognitive factors—among them, brand and type of product—have an effect on the perceived healthiness of food, but such features do not influence the choice and intake of food. The reviews of Alba and Williams [15], and Krishna [16] highlight the topic that continues to be researched ever since, namely that the perceived healthiness of food has an effect also on the assessment of the taste of food (e.g., [17,18]). However, research attests that the perception of the healthiness of food is not influenced by one factor only, but by a combination of factors [19,20], so we can state that this topic is highly complex and important both for consumers and companies.

The aim of our literature review is to assemble earlier research and survey the factors that influence consumers in their perception of the healthiness of food.

## 2. Research Methodology

In an attempt to access the articles related to the perceived healthiness of food, we employed several search engines—Science Direct, MDPI, Emerald Insight, Wiley Online Library—in our literature analysis. In recent years, numerous review-type articles touching on the topic of healthiness have been published (e.g., [12,13,14,15,16,17,18,21,22]), but they only fleetingly mention the issue. The present literature review, however, specifically approaches the topic from the consumers’ point of view and so examines the factors which, according to research literature, influence consumer perception of the healthiness of food.

Between 2012 and 2016, several review articles touched on the topic of perceived healthiness of food [13,15,16,21,22] or chose it as their main topic [14]. However, it has remained a widely researched area ever since, so we focused on the time period that followed. Articles published between 1 January 2014 and 31 March 2019 were selected using the following terms:

- “perceived healthiness of food”
- “evaluating food product healthfulness” OR “evaluation of food healthiness”

We looked for the terms in the title, the abstract or among the key words; naturally, because of the way they work, there were slight differences when using the different search engines.

In the I. case, on the ScienceDirect surface we looked for the exact term “perceived healthiness” in quotation marks in the “title, abstract or keywords” fields, while “food” appeared in the “terms” field. On the MDPI page, a very similar method was used, “perceived healthiness”—again in quotation marks—was searched for in the abstract, while “food” was searched for in “all fields”. Between the two terms specified in quotation marks, we used the AND relationship to make sure that the search results include both terms. On the Emerald Insight surface, we looked for the complete terms in the abstract and the title, while with Wiley Online Library, in the abstract only, without quotation marks.

**Figure 1:**
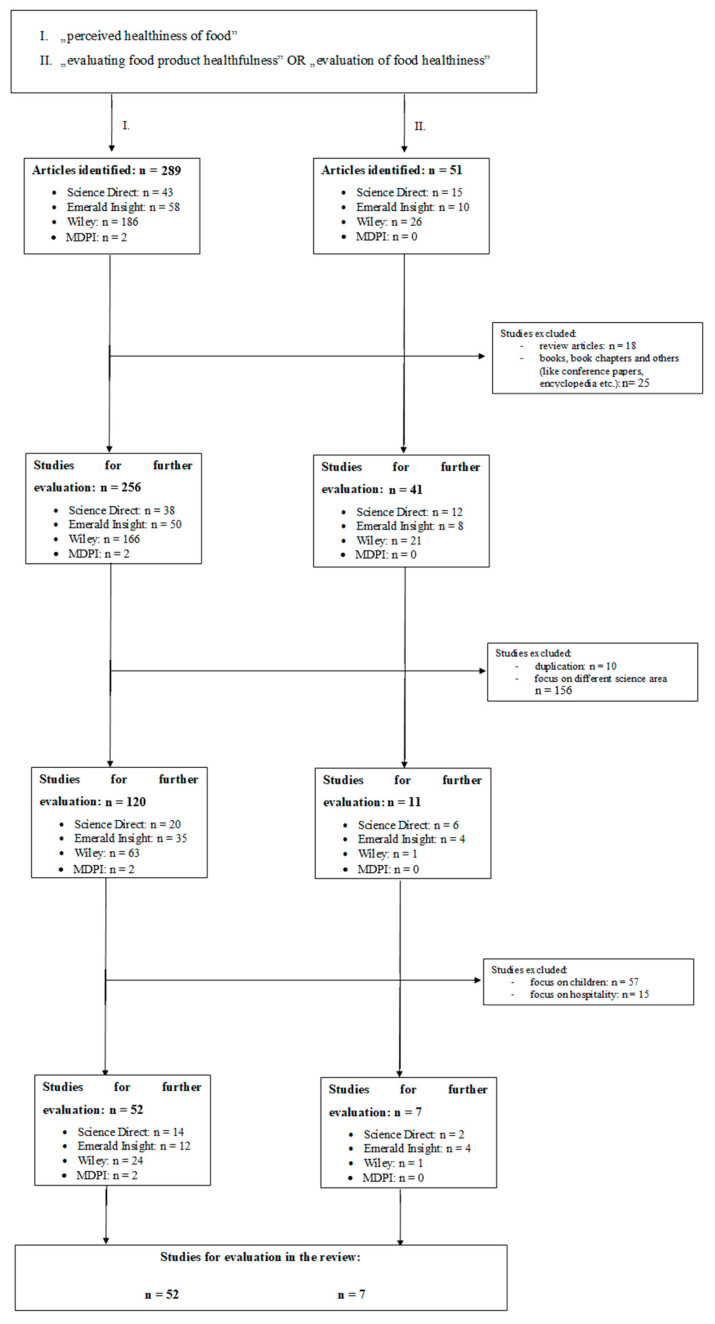
The search hits and the steps of their filtering.

In the II. case, on the ScienceDirect search field first “evaluating healthiness”, then “evaluation of healthiness” in quotation marks was in the “title, abstract or keywords” field, while “food” was in the terms field. Very similarly to this and point I, on MDPI, the previously mentioned terms were searched for in the abstract, while the term “foods” was searched for in all fields. Just like in the first case, we ran the search with the AND relationship between the search terms. With Wiley and Emerald Insight, we collected the articles in a similar way, looking for the terms in the abstract only and in the title and the abstract, respectively. The search results and the filtering of hits are illustrated in Figure 1.

In our analysis, we specifically focused on the products of the food industry, so we did not include research on restaurants, catering establishments, and those on various casseroles, boiled and fried foods served on plates. Moreover, articles on children’s dietary habits and on healthy food provision were also not included. The accessed full-length articles were evaluated by two authors (B.P. and Á.T.). Any contested issues were resolved by three authors (B.P., Á.T. and Z.L.).

## 3. Results

The main question of our research is what influences consumers in their perception of the positive effects of a given product on health, which, for the sake of simplification, we will refer to as the healthiness of the product. We provide a comprehensive display of the main results of the articles on the topic in Table 1, then we analyse them, reviewing the points of agreement and opposition.

Table 1 clearly shows that numerous factors influence consumers when assessing the healthiness of a product. In our literature analysis, we categorized these factors as follows:

- Communicated information [26,27,34,35,37,47,60,62,65,73,74,77];
- The shape and colour of the product packaging [23,25,28,32,33,43,61,66,70,71];
- The ingredients of the product [24,29,39,40,49,57,64,76];
- Product category [54,61,69,70,75,78];
- Organic origin of the product [5,31,42,45,46,48,51,55,68];
- The taste and other sensory features of the product [41,44,52,70,79].

**Figure 2:**
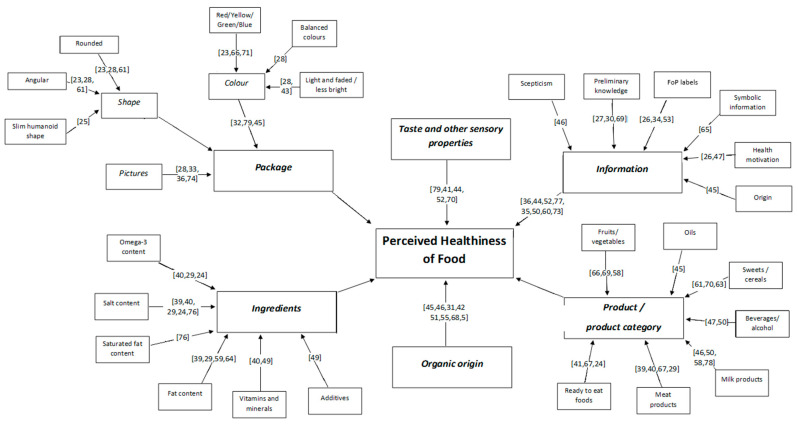
Factors influencing perceived healthiness.

The perceived healthiness of a food product is influenced by numerous factors. For bigger clarity, the main points of the research results are summarized in Figure 2.

### 3.1. The Effect of the Communicated Information on the Perceived Healthiness of a Product

When companies provide consumers with information related to nutritional value or to health effects in some way on the product packaging, it has a positive effect on perceived healthiness [26,34,35,36,47]. At the same time, care must be taken that consumers comprehend this information correctly, so that they do not evoke undesired associations [62], and consumer scepticism related to health claims must also be taken into account [74], as in this case information may even have a negative effect on assessing healthiness.

All this also entails how much of the messages communicated through the product a consumer will comprehend and thus how healthy they will perceive the product. This is supported by the previous knowledge of the consumer, which influences perceived healthiness to a great extent [27,69,78]. Moreover, perceived healthiness of a product is further improved by adding a picture of the product to the communicated information [36] and is also affected by FoP labels and health claims [26,34,38,53]. Although several studies show that FoP labels help consumers choose healthier foods [26,34], due to the diversity of FoP labels, it cannot be clearly stated that their use always helps greatly in increasing the perceived healthiness of the product [53].

The health motivation of the consumer also influences the assessment of the product; Machín et al. [26] maintain that it plays a pivotal role in the way front of package information is used. In contrast, according to the results of Rebouças et al. [47], consumer interest in healthy nutrition does not influence the acceptance and perceived healthiness and nutritional value of the product they examined (“cashew nut beverage”).

### 3.2. The Influence of the Shape and Colour of the Product Packaging

Research results confirm that the shape and colour of the product packaging influence the perceived healthiness of the product, but from certain aspects the results contradict each other. Whereas Marques et al. [23] maintain that a product is perceived to be healthier in a rounded packaging, other researchers [28,61] claim that consumers perceive a product healthier in angular shaped packaging. A further result related to the shape of the packaging is that packaging resembling a slim human figure is perceived healthier [25].

The influence of the colour of the packaging has also been examined by several researchers. According to the results of Marques et al.’s research [23], buttered products were perceived healthier in a red and yellow coloured packaging. The effect of the colour red is mentioned by several other studies. Wąsowicz et al. [66], along with yellow, green and blue, mention the colour red as a colour referring to health. At the same time, Reutner et al. [71] assert that the colour red can have a significant effect on the refusal of unhealthy foods. Certain colours and hues, however, can imply that the product is less healthy: research participants considered dark glass [45] and colours hinting at artificiality (“heather”, “pink”, “celadon”) [66] to be referring to unhealthy or less healthy products.

Contrasts resulting from the perception of colour can be attributed to the variety of products and their different packaging investigated in the studies; so it is possible that in the case of a buttered product, the red & yellow colour combination found by Marques et al. [23] was perceived healthier, while it can be different for other products. Therefore, when discussing the effect of colour, it is important to acknowledge the influence of product category. Differences between countries are also important; so for example, in Denmark, paler, whereas in the United States, balanced colour tones are more standard on healthy products [28]. Moreover, the effect of colour can differ according to the age of the consumer: for example, with young people, colours have a stronger effect than health messages [32].

### 3.3. The Effect of the Ingredients of a Product on its Perceived Healthiness

Results related to ingredients show that consumers mostly pay attention to the ingredients that nutrition experts emphasize in relation to healthy nutrition. The majority of the research studies we have examined address sodium- and fat content as well as omega-3 content. According to the results of Lazzarini et al.’s [59] research, the fat content of a product is an indicator of perceived healthiness for consumers.

Whether it is Bolognese sauce, frankfurter sausages, or other processed meat products, consumers prefer a reduced sodium- and fat content, so that is how a company can make consumers perceive these products healthier [24,29,39]. Moreover, while there are consumers who rely on the fat- and fibre content of the product for its perceived healthiness [64], others ignore the protein-, sodium-, and saturated fat content when making decisions [76].

The other ingredient featuring in numerous studies was omega-3. In several cases, consumers would choose to change an ingredient other than the fatty acid [24], or they did not consider the product suitable for the addition of omega-3 [29]; at the same time, they prefer if omega-3 fatty acid is added to the product rather than if nothing is added [40].

### 3.4. The Effect of Product Category on Perceived Healthiness

Perceived healthiness is also influenced by the product or product category [75,78]; in fact, in Orquin’s [78] research, product category emerged as one of the two main factors based on which consumers perceived the healthiness of a product. In the studies, products assigned to different categories according to different criteria were compared. Fenko et al. [61] compared consumer perception of cereal- and buttered cookies, of which consumers perceived cereal cookies healthier. Vasiljevic et al. [70] compared muesli bars and chocolate, and their results show that, regardless of label, consumers perceived chocolate tastier and muesli bars healthier. According to Maehle et al.’s [75] surprising result, consumers are less concerned about the healthiness of the product if they consume it for the nutritional value (utilitarian products), than in the case of products consumed for pleasure (hedonic products).

### 3.5. The Effect of Organic Origin on Perceived Healthiness

Results of numerous studies have confirmed a positive effect of organic origin on the perceived healthiness of a product [42,45,48,51,68]. In addition, organic origin also facilitates the understanding of the communication of “healthy food” [5]. Health-conscious consumers also tend to show openness towards bio foods and generally ignore the health-related messages of functional foods [46].

### 3.6. The Effect of the Sensory Features of the Product on Perceived Healthiness

The sensory features of the product also play a role in its perceived healthiness. The taste and other sensory features of the product may dominate over the perception of healthiness [41,52,79], and if the sensory features of the product do not satisfy the consumer, then communicating the nutritional value is not enough to make the product accepted [44].

## 4. Discussion

The aim of our literature analysis was to explore the factors that influence the perceived healthiness of food products. Numerous studies set out to discover what influences the perceived healthiness of individual products in the time period we focused on. In the present article, we only considered research results related to foods.

Based on the research results, we identified six categories that influence perceived healthiness of a product: the effect of the communicated information, product category, the shape and colour of the product packaging, the ingredients of the product, the organic origin of the product and the taste and other sensory features of the product.

The effect of the communicated information clearly influences perceived healthiness; at the same time, previous knowledge clearly affects how this information influences perception. Product category is a main factor in the perceived healthiness of a product. In recent years, a diverse range of product categories has been tested, which makes generalizations difficult.

The most numerous contradictory research results were related to the shape and colour of the product packaging, which calls for further investigation. Research results are ambiguous concerning whether angular or rounded packaging is more suitable to communicate healthiness. One of the most researched colours is the colour red. Nevertheless, results related to the colour red do not point in the same direction.

Research results related to the ingredients of the product confirm that reducing the sodium-, sugar- and fat content increases consumer acceptance of the improved product in terms of health; at the same time, research does not give a definite answer regarding consumer perception of the possible enriching ingredients.

The organic origin of the product positively influences perceived healthiness. Health halo effect emerged in several studies in connection with bio products.

Basically, the taste and other sensory features of the product dominate over perception of healthiness. A common result of the examined studies showed that the unsuitability of sensory features cannot be balanced out by favourable perceived healthiness.

Our collected results and their juxtaposition can help with the proper planning of product development and marketing communication, and they also raise further research questions related to the inconsistent results. Our conclusions can serve as a baseline from several aspects when devising packaging of a new product. They can help with the proper design of the packaging, both in terms of shape and the used colours, and with choosing the right FoP labels. The choice of the labels used on the packaging requires special care. The type of health claim communicated by the company has to be considered carefully, provided that the use of a health claim is effective in the first place. At the same time, it is also important to consider that communicating the different ingredients may be an effective method to reach its goal.

Within the categories, we have found several conflicting results, as well as unanswered research questions, which call for further research. The most important aim of further research may be to gauge the effect of the discovered aspects relative to each other, even comparing all the aspects.

Further research may also aim at clarifying the emerged controversial results, as the research results are not uniform for example in connection with the shape of packaging and colours that evoke a healthy feeling, keeping in mind that these factors may change according to product category. Apart from clarifying discrepancies, further research may take on the task of testing specific features on different food products. Treating the constructed system as a complex entity, it is worth examining whether a different colour and shape of packaging is justified to communicate the health benefits of each food.

## 5. Limitations

In the course of our literature analysis, we encountered several barriers that have to be taken into account when evaluating the results. There has not been a review article on the topic since 2016, even though several new studies have been published since then. As we reviewed only the 2014–2019 time period, we can only report the results of the most modern research. The surfaces used for data collection are also important to mention: during the research, we had no access to the surfaces covering the whole literature, therefore we chose the above described search surfaces, where we could access the full-length articles. Our options were limited by the year-to-year changes in the agreement between the Hungarian Electronic Information Service National Programme (EISZ) and Elsevier [81].

## Figures and Tables

**Table 1:** The articles included in the literature analysis and their main Claims.

Source	Year	Country	Method	Item Number	Main Claims
Marques da Rosa, Spence and Miletto Tonetto [23]	2019	Brazil	2 × 3 within-groups experimental design; 2 × 2 × 2 intra-groups experiment design	50 + 102	A buttered product was considered healthier in a round, red and yellow packaging The colour and shape of the packaging influence perceived healthiness
Pires, de Noronha and Trindade [24]	2019	Brazil	Online survey; focus groups	263 + 16	In the case of Bolognese sauce, consumers prefer less sodium to omega-3 content
Yarar, Machiels and Orth [25]	2019	Not indicated	One factorial between subject design	78 + 144	Consumers consider a product in packaging that resembles a slim human figure healthier, especially if they themselves do not have such a figure The shape of the packaging plays an important role in the perceived healthiness of the product
Machín et al. [26]	2018	Uruguay	shopping situation (on online surface)	1182	“FOP nutrition labelling schemes effectively improved the average healthfulness of food choice by respondents.” (p. 60) Health motivation can play a key role in the use of FOP (front of package) nutrition information
Hartmann et al. [27]	2018	UK, Sweden, Poland, France	Online survey	1950	Indicators of perceived healthiness: searching for information, knowledge on nutrition, and the health effects of the nutrients. There was a willingness to pay extra for “Free-from” products among those who look for information and prefer natural products.
Festila and Chrysochou [28]	2018	Denmark, United States	Content analysis	2545 products	The colour, shape, material of and the illustrations on the packaging differ between the products claimed healthy and those considered “normal” in general and also according to product category Products considered healthier appear on the market with lighter, matter or more balanced colours and in angular packaging in the examined countries
Polizer Rocha et al. [29]	2018	Brazil	Word association test, EsSense profile, attitudinal questionnaire	120	The biggest health advantage for consumers of frankfurters can be achieved through a decreased sodium- and fat content. Omega-3 and fibre source are less preferred features in this product
Wijayaratne et al. [30]	2018	Australia	Two stage online survey	756	Food-literacy has a positive effect on the attitudes of the “dietary-gatekeepers” consumer group towards healthy food Those with higher food-literacy are more confident in the preparation of a healthier diet
Lee et al. [31]	2018	Taiwan	Survey	122	Although a bio label influences perceived healthiness, it does not increase the consumption of such products among “health externals”
Vila-López and Küster-Boluda [32]	2018	Spain	Experimental sessions	300	Younger consumers are more influenced by aesthetic/commercial signs (colours) than by “technical cues” (healthy messages)
Lidón et al. [33]	2018	Spain	between-subjects experiment	147	Placing a picture suggesting healthiness on the packaging may increase willingness to purchase When perceiving a product, there is a strong positive relationship between healthiness and product quality
Acton and Hammond [34]	2018	Canada	Online survey	1000	A small group of the respondents (5–10%) said that the “high in…” caption in the front of the packaging (FOP) seemed harsh for them According to the majority of the respondents, FOP captions help to better control the choice of healthy food products
Carabante et al. [35]	2018	USA	Consumer test, questionnaire	150	Communicating the health benefits of the fat composition resulting from the diet of grass-fed beef increased overall liking and purchase intent “Health Benefit Information” (HBI) decreased the effect of juiciness and tenderness on overall liking
Miraballes and Gámbaro [36]	2018	Uruguay	Conjoint analysis	60 + 60	A product was considered healthier if, in addition to the caption communicating ingredients, there was also a picture/image on it
Wardy et al. [37]	2018	USA	Consumer testing	128	A 50% and/or 100% decrease of saccharose and the communication of this fact—displaying HBI- had a positive effect on the overall liking of the product
Benson et al. [38]	2018	Ireland	Survey	1039	Respondents rated the healthiness of the tested products the same regardless of the “nutrition and health claims”, there was no significant difference in their assessment
Shan et al. [39]	2017a	Republic of Ireland	Focus groups	40	The perception of consumers was influenced by the healthiness, taste, and prevalence of the product To make processed meat products healthier, participants would decrease the sodium- and fat content rather than add health-preserving ingredients
Shan et al. [40]	2017b	Republic of Ireland	Survey	481	Participants preferred enrichment with omega-3 to the non-enriched product, and the least preferred enriching ingredient was vitamin E.
Labbe et al. [41]	2017	Switzerland	Conjoint	57	The choice among frozen pre-packaged pizzas was more influenced by the expected taste experience than by perceived health effect and was not influenced by the expected feeling of being sated.
Prada et al. [42]	2017	Portugal	Survey	204 + 85	Products of organic origin were considered healthier, tastier and less energy-filled than their traditional counterparts-– “halo-effect” in case of bio food products
Tijssen et al. [43]	2017	Netherland	Experiment; Implicit Association Test (IAT)	148 + 140	Participants associated paler coloured packaging with health, whereas regular packaging was considered more striking Wrapping a ‘healthier’ product in warmer, fuller, pale coloured packaging improves sensory expectations, and can make the product more attractive
Marino et al. [44]	2017	Italy	Sensory analysis and consumer survey	8 + 250	When choosing healthy food products, the expected less good taste is the biggest obstacle for consumers not wanting to forgo good taste If the sensory features of a product are not appropriate, information on nutritional characteristics is not enough for the consumers to choose healthier alternatives
Cavallo and Piqueras-Fiszman [45]	2017	Italy, Netherlands	Consumer survey (online questionnaire)	214	Italian origin played the biggest role in the perceived healthiness of the examined product (olive oil) Having a bio origin positively influenced perceived healthiness For Dutch consumers, hot taste had a negative influence on perceived healthiness, whereas Italian consumers were not influenced by it In general, a darker glass bottle had a negative effect on the perceived healthiness of the examined product, with some exceptions: it had a positive influence on Italian consumers and on those for whom the origin of the product is important
Gineikiene, Kiudyte and Degutis [46]	2017	Lithuania	Survey; Structural equation modeling	295	Health-conscious consumers tend to disregard messages related to the health benefits of functional foods, and prefer bio food products In the case of functional, organic, and traditional products, scepticism towards health claims has a stronger negative effect on the perceived healthiness than the effect of health consciousness
Rebouças et al. [47]	2017	Brazil	Sensory evaluation	96	Information on the ingredients and nutritional values of cashew- and soy drinks and functional statements related to this information have a positive effect on consumers’ perception of healthiness and of nutritional values The extent of consumer attention paid to a healthy diet and food neophobia did not influence perceived healthiness of the product.
Tleis, Callieris and Roma [48]	2017	Lebanon	Face-to-face survey	320	Lebanese consumers purchase bio- products because they consider them healthier and safer
Brečić, Mesić, and Cerjak [49]	2017	Croatia	Face-to-face interviews	500	The dominant factor explaining 18.8% of the sample is “health and sensory characteristics”. The factor includes the sensory characteristics (taste, smell) of the product and its composition One segment is the “healthy and tasty food lovers” who are sensitive to the “inner” characteristics of the food: they are concerned about additives and artificial ingredients and prefer foods rich in vitamins and minerals
Thomson et al. [50]	2017	Melbourne, Shanghai, Vietnam, Indonesia, Singapore	Online survey	3951	there are differences in the perceived healthiness of a certain product between respondents from different countries sweetened, higher circulation products and children’s drinks were considered healthier in Vietnam, Shanghai and Indonesia than in Singapore and Melbourne
Apaolaza et al. [51]	2017	Spain	one-way between-groups experimental design	90	“the organic halo effect on hedonic evaluation and purchase intention was totally mediated by increases in sensory ratings and perceived healthiness, providing a process explanation for this effect” indicating the organic origin of the product significantly increased its perceived healthiness
Anders and Schroeter [52]	2017	Canada	Survey	8114	Taste, convenience and affordability are more important than information related to healthiness and the resulting benefits
Talati et al. [53].	2016	Australia	Survey	2058	Testing different FoP labels and their effect on perceived healthiness “daily intake guide” and “multiple traffic light” had a positive effect on the global perception of the product compared to when no FoP labels were used Nevertheless, FoP labels only had a weaker effect on perceived healthiness, but a bigger impact on global evaluations
Samoggia [54]	2016	Italy	Face-to-face survey	402	Health-oriented consumers are open to health-enhancing wine products, and their willingness to pay is also higher. Consumers of wine think that consumption of wine offers protection against hypertension and atherosclerosis. Consumers consider wine a healthy product
Seegebarth et al. [55]	2016	USA, Germany	Survey	206 + 240	American consumers appreciated the functional values provided by bio foods more than German consumers did. Moreover, American consumers purchase bio food because they consider them healthier and of better quality.
Puska & Luomala [56]	2016	Finland	Pilot test + online survey	17 + 1081	Respondents expect different health benefits from two products perceived equally healthy (“physical well-being, outward appearance, energy dimensions” vs. “emotional well-being, self-management and social responsibility”)
Larkin and Martin [57]	2016	UK	Experimental sessions	141	The weight of the consumer influences their perception of the calorie content of a product considered healthy, while this effect is less pronounced in the case of “unhealthy” food Consumers underestimate the calorie content of foods considered healthy compared to those considered unhealthy
Szocs and Lefebvre [58]	2016	USA	Within subjects experiment, lab study, between subject design,	122 + 111 + 166	Perceived healthiness and perceived calorie content are not influenced by the physical state of the product (e.g., liquid or solid) Participants perceived more processed products less healthy and richer in calories Participants considered the less processed fruit and yoghurt plate healthier than the more processed smoothie
Lazzarini et al. [59]	2016	Switzerland	Experiment	85	The perceived healthiness and the perceived environmentally friendly nature of a product correlate The indicators of perceived healthiness: product category, fat content, extent of processing and the indication of organic origin
Jo et al. [60]	2016	France	Framed field experiment	129	Consumers are willing to pay more for “healthy” products if objective information on the nutritional composition is available Information on nutritional value increases willingness to pay for “healthy” foods, while decreases it for foods considered unhealthy
Fenko, Lotterman and Galetzka [61]	2016	Netherlands	Questionnaire	165	Products in angular packaging were perceived healthier than those in rounded packaging The higher a consumer’s general health interest, the less they considered a product healthy Product category significantly influenced perceived healthiness, while brand name did not
Hipp et al. [62]	2016	USA	Survey	2015	The examined signs and symbols that were displayed on vending machines and at cafés in order to foster health-conscious food choices did not help consumer decision
Rizk & Treat [63]	2015a	USA	Survey	272	In the case of products in bigger packaging/portions participants had difficulty in distinguishing their perceived healthiness
Rizk and Treat [64]	2015b	USA	Survey	169	Single women mostly relied on fat- and fibre content when assessing the healthiness of a product Displaying protein- and sugar content mitigated reliance on fat- and fibre content
Sütterlin and Siegrist [65]	2015	Switzerland	Experiments	164 + 202 + 251 + 162	people assess the healthiness of a product with the help of simple heuristics—e.g., in the case of fructose: fruit-healthy—see health halo effect
Wąsowicz et al. [66]	2015	Poland	Focus group, survey	8 + 90	consumers associate certain colours with the healthiness of the product. yellow, blue, red and green colours may indicate healthiness blue and yellow colours evoked positive emotions both from the perspective of healthiness and of naturalness
Luomala et al. [67]	2015	Finland	Personal and group interviews	40	The dieting status and health motivation of consumers as well as the assessment of the benefits offered by the product influence the perceived taste and healthiness of the product Those who are not on a diet are more critical in their assessment of what is tasty and healthy Those on a diet consider light salad dressing and light sausage healthy, while those not on a diet consider these products unhealthy
Xie et al. [68]	2015	China	Survey (questionnaire) + in depth interviews	388 + 18	Health benefits are one of the most important factors that make consumers purchase organic products
Grubor et al. [69]	2015	Serbia	Focus groups, survey	? + 300	“Consumers’ health attitudes” mostly influence the consumption of enriched products the pre-enrichment version of which they had already been familiar with
Vasiljevic, Pechey, and Marteau [70]	2015	UK	Between-subject experiment	955	Regardless of the label, participants considered chocolate tastier, and a muesli bar healthier A frowning emoji on a white background had the effect of a muesli bar being considered less tasty and less healthy Emojis had a stronger influence on the perception of healthiness and tastiness of snacks than did coloured labels Frowning emojis have a stronger influence than smiley ones on perceived healthiness for products where perception of healthiness is influenced by the health halo effect
Reutner, Genschow and Wänke [71]	2015	Switzerland	Between subject experiment	91 + 143	The colour red influences the assessment of products considered unhealthy (dangerous) more than that of healthy products Using red colour mitigated the consumption of foods considered unhealthy, and also influenced the choice of these products
Thomsen and Hansen [72]	2015	Denmark	qualitative pilot study; survey	16 + 599	Improving consumer knowledge on healthy nutrition could help to make healthy food choices It is difficult to improve the knowledge of consumers who take less interest in healthy nutrition
Dharni and Gupta [73]	2015	India	Survey	150	Perceived usefulness of nutritional information is of key importance when making decisions related to healthy nutrition Understanding information increases perceived usefulness, while the increase of perceived usefulness facilitates choosing better- healthier- food
Annunziata, Vecchio and Kraus [74]	2015	Italy	Survey	400	Consumers over 60 are influenced by health claims in the assessment of the healthiness of functional foods Consumers over 60 have difficulty verifying the reliability of information Among the several used symbols, heart was the most valuable for elderly consumers
Maehle et al. [75]	2015	USA	Conjoint analysis	306	The issue of healthiness is less important in the case of “utilitarian food products” than for hedonic foods Moreover, in the case of “utilitarian food products”, the healthiness of the product is the least important feature compared to the taste and price of the product and the usage of “environmental label”
Bucher, Müller & Siegrist [76]	2015	Switzerland	Survey	85	Lay consumers assessed the healthiness of a product according to aspects similar to those of experts’ When making decisions, lay consumers ignored the quantity of saturated fat, protein, and sodium in the product Lay consumers were quite able to assess the nutrition profile of individual food items, but were less able to do so with complete dishes
Kraus [77]	2015	Poland	Survey	200	The most important health-related features can be ranked the following way: (1) strengthens the immune system (2) lowers the risk of tumour-related diseases (3) lowers the risk of cardiovascular diseases
Rodman et al. [78]	2014	USA (Baltimore, Maryland)	In-depth interview	36	Organic origin is important for consumers when assessing the healthiness of a product. When communicating the healthiness of the product, organic origin can have effectiveness similar to other health messages.
Orquin [79]	2014	Denmark	Brunswik lens model	1329	Perceived healthiness mainly depends on two factors: product category and consumer knowledge on individual products Consumers underestimate the healthiness of milk and yoghurt and overestimate that of butters and cheeses Consumers are inclined to perceive a product healthier if they are familiar with it
Carrete and Arroyo [80]	2014	Mexico	In-depth interviews, focus groups	8 + 30	In general, the taste, colour, and texture of a product are more important for consumers than nutritional characteristics, which hinders healthier nutrition
Lin [81]	2014	Taiwan	2 × 2 experimental design	170 + 177	happier people are more variety seeking in the case of healthful products or products they are not familiar with, while sadder people are more open to variety in the case of hedonic or familiar products The type of the product “(hedonic vs. Healthful products)” influences the relationship between variety seeking and the mood of the consumer
